# Changes in Tumor Biology During Chemoradiation of Cervix Cancer Assessed by Multiparametric MRI and Hypoxia PET

**DOI:** 10.1007/s11307-017-1087-5

**Published:** 2017-05-24

**Authors:** Petra Georg, Piotr Andrzejewski, Pascal Baltzer, Michaela Daniel, Wolfgang Wadsak, Markus Mitterhauser, Alina Sturdza, Katarina Majercakova, Georgios Karanikas, Richard Pötter, Marcus Hacker, Thomas Helbich, Dietmar Georg, Katja Pinker

**Affiliations:** 1 Department of Radiation Oncology, Medical University of Vienna/AKH Wien, Währingergürtel 18-20, 1090 Vienna, Austria; 2 Christian Doppler Laboratory for Medical Radiation Research for Radiation Oncology, Währingergürtel 18-20, 1090 Vienna, Austria; 3 Department of Biomedical Imaging and Image-guided Therapy, Division of Molecular and Gender Imaging, Medical University of Vienna/AKH Wien, Währingergürtel 18-20, 1090 Vienna, Austria; 4 Department of Biomedical Imaging and Image-guided Therapy, Division of Nuclear Medicine, Medical University of Vienna/AKH Wien, Währingergürtel 18-20, 1090 Vienna, Austria; 5 CBmed - Center for Biomarker Research, Stiftingtalstrasse 5, 8010 Graz, Austria; 6 Ludwig-Boltzmann-Institute for Applied Diagnostics, Währingergürtel 18-20, 1090 Vienna, Austria; 7 Department of Radiology, Memorial Sloan-Kettering Cancer Center, 1275 York Avenue, New York, NY 10065 USA

**Keywords:** Multiparametric MRI, PET, Hypoxia, Cervix cancer, Response assessment

## Abstract

**Purpose:**

Imaging biomarkers assessed with magnetic resonance imaging (MRI) and/or positron emission tomography (PET) enable non-invasive tumor characterization in cervix cancer patients. We investigated the spatio-temporal stability of hypoxia, perfusion, and the cell density of tumors over time by repetitive imaging prior to, during, and after radio-chemotherapy.

**Procedures:**

Thirteen patients were included in this prospective study. The imaging protocol included the following: [^18^F]fluoromisonidazole ([^18^F]FMISO)-PET/x-ray computed tomography (CT) and multiparametric (mp)-MRI at four time-points (TP): baseline (BL); and weeks 2 (TP1), 5 (TP2), and 19 after treatment start (follow-up FU). Complete datasets for six patients could be assessed for tumor volume, enhancement kinetics, diffusivity, and [^18^F]FMISO-avidity (P1–P6). In addition, two patients completed all PET/CT examinations (P7–P8) but not all MR scans; however, one of them had no hypoxia (P8). Descriptive statistics, correlations, and voxel-by-voxel analysis were performed. For various, independent reasons, five patients could not complete the study according to the protocol with all imaging sequences.

**Results:**

Median tumor ADCs (in ×10^−3^ mm^2^/s) were 0.99 ± 0.10 at BL, 1.20 ± 0.12 at TP1, 1.33 ± 0.14 at TP2, and 1.38 ± 0.21 at FU. The median TBR_peak_ (tumor-to-background) was 2.7 ± 0.8 at BL, 1.6 ± 0.2 at TP1, 1.8 ± 0.3 at TP2, and 1.7 ± 0.3 at FU. The voxel-by-voxel analysis of the [^18^F]FMISO uptake at BL and TP1 showed no correlation. Between TP2 and TP1 and FU and TP2, weak correlations were found for two patients.

**Conclusions:**

Longitudinal mp-MR and PET imaging enables the *in vivo* tumor characterization over time. While perfusion and cell density decreased, there was a non-uniform change of hypoxia observed during radiotherapy. To assess the potential impact with regard to more personalized treatment approaches, hypoxia imaging-based dose painting for cervix cancer requires further research.

## Introduction

Advanced imaging methods have been explored extensively for tumor and tissue characterization in the light of individualized precision radiation oncology. The potential of these methods to visualize and quantify the characteristics of tumor subvolumes has been demonstrated in response assessment studies and in studies quantifying tumor aggressiveness [1–3]. With parameters derived from dynamic contrast-enhanced magnetic resonance imaging (DCE-MRI) poorly perfused regions within cervical cancer tumors could be determined and were shown to be independent predictors for recurrence and death [4–6]. Diffusion-weighted MRI (DWI) allows insights into tissue microstructure, membrane integrity, and cell density, and deduced imaging biomarkers were successfully explored for response assessment of cervical cancer [7, 8].

Depending on the radiopharmaceutical applied, positron emission tomography (PET) can provide different biological information, *e.g.*, about tumor metabolism or the presence of hypoxia [9–11]. For hypoxia imaging, [^18^F]fluoromisonidazole ([^18^F]FMISO) has become a frequently used radiotracer. PET imaging with this tracer has proven to be useful in the assessment and prediction of the outcome of different types of cancer therapy [12, 13]. Hypoxia is known to cause radiation treatment resistance and therefore new therapeutic strategies are targeting this crucial tumor feature. The clinical implementation of dose escalation to sub-target volumes is challenged by their temporal dynamics. Several studies suggest that hypoxia may vary during the course of treatment [14–18]. A recent study based on [^18^F]fluoroazomycin arabinoside ([^18^F]FAZA) for hypoxia imaging in head and neck cancer and lung cancer patients described a relatively stable tumor hypoxia only at week 2 after the start of treatment [19]. Another study, correlating quantitative imaging analysis with immunochemistry, concluded that various methods of [^18^F]FMISO PET analysis perform differently for the assessment of tumor hypoxia [20]. Consequently, baseline imaging might not be sufficient for dose escalation studies driven by hypoxia imaging.

The aim of this exploratory study was to investigate the spatio-temporal stability of non-invasively measured tumor hypoxia, perfusion, and cellular density of cervix cancer with multiparametric (mp)-MRI and [^18^F]FMISO PET/x-ray computed tomography (CT) by repetitive imaging prior to, during, and after completing chemoradiation.

## Material and Methods

### Patients

Thirteen patients were included in this Institutional Review Board (IRB)-approved, prospective, single-institution study. The imaging protocol included 2-deoxy-[^18^F]fluoro-D-glucose ([^18^F]FDG) PET/CT prior to treatment and [^18^F]FMISO PET/CT as well as mp-MRI at four time-points (TP): at baseline (BL) and in weeks 2 (TP1), 5 (TP2), and 19 after the treatment start (follow-up, FU), for details see section below. All thirteen patients fulfilled the following inclusion criteria: ≥18 years, histological confirmed cervical cancer, and no contraindications to contrast agents. All patients gave written informed consent.

The treatment included definitive concurrent chemoradiotherapy (cisplatinum 40 mg/m^2^ weekly for 5 weeks) with intensity-modulated radiotherapy (IMRT) followed by advanced brachytherapy [21]. The IMRT dose prescription was 45 Gy in 25 fractions. In weeks 6 and 7, MRI-guided high dose-rate brachytherapy (HDR-BT) with combined intracavitary/interstitial applicator was performed. The dose prescription was 28 Gy in four fractions to the high-risk clinical target volume (HR-CTV) [22].

Table 1 lists the 13 patients and their characteristics including clinical response after therapy where available, and the respective imaging data sets that could be acquired. Six patients completed the study according to the protocol with all imaging sequences. In addition, two patients completed all PET/CT examinations (P7–P8). One of these patients refused MR scans at TP1 and TP2 due to poor general condition after chemotherapy; the other could not undergo MRI after BL scan any longer due to surgical clip after rectum polypectomy. In the latter patient, no hypoxia was detected at any time-point and thus she was not included in further evaluation.

**Table 1:** Patient characteristics—clinical status before and after treatment

Patient	Age	FIGO Stage	Outcome		BL		TP1		TP2		FU	
					MISO	MRI	MISO	MRI	MISO	MRI	MISO	MRI
P1	39	IIB	CCR	NED	✔	✔	✔	✔	✔	✔	✔	✔
P2	55	IIB	CCR	NED	✔	✔	✔	✔	✔	✔	✔	✔
P3	59	IVB	LR, RR, DM	AWD	✔	✔	✔	✔	✔	✔	✔	✔
P4	57	IIIB	DM	DOD	✔	✔	✔	✔	✔	✔	✔	✔
P5	37	IVB	CCR	NED	✔	✔	✔	✔	✔	✔	✔	✔
P6	66	IVB	DM	DOD	✔	✔	✔	✔	✔	✔	✔	✔
P7	36	IIB	CCR	NED	✔	✔	✔		✔		✔	✔
P8^a^	60	IIB	CCR	NED	✔	✔	✔		✔		✔	
P9	61	IIB	LR	DOD	✔	✔	✔	✔	Consent withdrawn			
P10	73	IVB	CCR	DUR	✔	✔	✔	✔	Consent withdrawn			
P11	58	IIB	No follow-up		✔	✔	Treatment interrupted					
P12	49	IVA	No follow-up		✔	✔	Treatment interrupted					
P13	45	IIB	No follow-up		✔	✔	Treatment interrupted					

*CCR* continuous complete remission, *LR* local recurrence, *RR* regional recurrence, *DM* distant metastases, *NED* no evidence of disease, *AWD* alive with disease, *DOD* dead of disease, *DUR* dead of unknown reason

^a^Patient with no hypoxia detected

The other five patients did not complete the imaging study at all time-points for the following reasons: three did not complete the full radiotherapy treatment (IMRT + HDR-BT) and two withdrew their consent after imaging at TP1. Patients with incomplete data sets were evaluated separately for feasibility purposes, but were not included in the analysis.

### Radiotracer Preparation

Radiotracers were produced using the fully automated FASTlab platform (GE Healthcare) with GMP compliant single-use cassettes in accordance with national health legislature guidelines. Synthesis protocols followed well-established procedures [23, 24].

### Imaging

All imaging studies were performed using a hybrid PET/CT system (Biograph 64 TruePoint PET/CT system, Siemens, Erlangen, Germany) and a 3 T MRI (TimTrio, Siemens, Erlangen, Germany) using a combination of eight-channel spine array coils (24 elements in eight clusters) and two-channel body array (six elements in two clusters) for signal acquisition.

For [^18^F]FDG PET/CT, patients fasted for 5 h and blood glucose levels were <150 mg/dl (8.3 mmol/l). All patients received a body weight-adapted injection of approximately 200–350 MBq [^18^F]FDG and [^18^F]FMISO on different days. Scanning in the supine position was started after an uptake time of 60 min for [^18^F]FDG and 210–240 min for [^18^F]FMISO. For both radiotracers, an unenhanced CT scan was recorded for attenuation correction; for [^18^F]FMISO, low dose scans were performed. Only [^18^F]FDG PET/CT was performed with i.v. contrast. PET images were reconstructed using the iterative TrueX algorithm (Siemens, Erlangen, Germany) [25, 26]. Four iterations per 21 subsets were used, with a matrix size of 168 × 168, a trans-axial field of view of 605 mm, and a section thickness of 3 mm. In the following study, only results of the repetitive [^18^F]FMISO PET datasets will be presented. Results on the correlation between [^18^F]FDG and [^18^F]FMISO at baseline have been described earlier [27].

All mp-MRI studies were performed with the patient in the supine position applying the MRI protocols listed in Table 2. Gadoterate meglumine (Gd-DOTA; Dotarem®, Guerbet, France) was injected intravenously as a bolus (0.1 mmol/kg body weight) using a power injector at 4 ml/s, followed by a 20-ml saline flush. The total MRI examination time was about 30 min.

**Table 2:** Summary of applied MRI protocols

Protocol	TR (ms)	TE (ms)	# of slices	FOV (mm)	AVR	Voxel size (mm3)	Scan time (min)	Comment
T2w TSE	4630	89	30	220	3	0.6 × 0.6 × 3.0	5:16	Axial
3D slab-selective T2w TSE	1500	173	176	300	2	0.9 × 0.9 × 0.9	3:56	Sagittal; SPACE
DWI	6300	82	30	280	5	1.5 × 1.5 × 5.0	3:28	Axial; 2D echo-planar with SPAIR fat suppression, *b* values 50 and 850 s/mm^2^ [28, 29]
T1w VIBE	3.38	1.38	52	380	1	0.8 × 0.8 × 3.0	0:53	Axial; SPAIR fat suppression; three repetitions: before, 1 min, and 4 min after contrast agent application
T1w TSE	675	12	30	280	2	0.6 × 0.6 × 3.0	4:19	Axial, fat suppression

*TR* repetition time, *TE* echo time, *FOV* field of view, *AVR* averages, *T2w* T2-weighted, *TSE* turbo spin echo, *SPACE* Sampling Perfection with Application optimized Contrasts using different flip angle Evolution, *T1w* T1-weighted, *DWI* diffusion-weighted imaging, *SPAIR* with spectrally adiabatic inversion recovery, *VIBE* volume interpolated breath-hold

### Image Fusion and Data Analysis

PET and MRI images were fused semi-automatically using registration tools of the treatment planning system RayStation (RaySearch Laboratories, Stockholm, Sweden) and were analyzed using the software Mirada RTx (Mirada Medical Ltd., Oxford, UK).

In brief, datasets acquired with the same scanners, *i.e.*, PET/CT and mp-MRI data, were initially fused according to DICOM tag information and the registration was corrected for possible patient movement. To assess the temporal and spatial changes in [^18^F]FMISO uptake, PET datasets from respective TP were deformably registered (TP1 to BL, TP2 to TP1, and FU to TP2). To avoid registration errors, *e.g.*, due to large volumetric changes, only directly neighboring time-points were evaluated. Registration between MRI and PET images was performed with an intermediate step, where CT (initially in the coordinate system of the PET) was deformably registered to the T2w MRI. The determined deformation field was subsequently used to deform the PET dataset (using an in-house developed Python code) to correlate MRI and PET data acquired at the same TP. In both cases, readers visually checked, in consensus, the correlation of the anatomy on respective modalities. CT-CT and MRI-CT registrations were performed using hybrid intensity/structure-based and structure-based only algorithms, respectively. For this reason, the whole uterus was defined on all CT and T2w MRI datasets. For the purpose of voxel-by-voxel analysis, all registered datasets were resampled to a common voxel size of 3 × 3 × 3mm^3^ using SlicerRT [28]. The Mirada RTx was applied only to handle data and to extract respective quantitative imaging parameters. Phython and Slicer RT were used to automate the routines performed in RayStation, to resample images, as well as to harmonize DICOM formats.

An experienced radiation oncologist (PG, >10 years clinical experience in cervix cancer), a radiologist (KP, >10 years clinical experience in MRI), and a nuclear medicine physician (GK >10 years clinical experience in PET) evaluated the non-processed data. In addition to the structures defined for registration purposes, the gross tumor volume (GTV) was contoured on the T2w MRI at BL, TP1, and TP2 to monitor the target shrinkage and extract quantitative image parameters (in FU the cervix structure was used to record image characteristics). At all four time-points, the following imaging parameters were extracted from the contoured GTVs:

1. DCE-MRI: semi-quantitative curve type analysis was performed to assess GTV’s enhancement kinetics in the early (1 min) and delayed phase (5 min), by calculation of the initial enhancement (IE) defined as (*I* ^DCE^_early_ − *I* ^DCE^_native_)/(*I* ^DCE^_native_) and wash-out rate (WO) defined as (*I* ^DCE^_delayed_ − *I* ^DCE^_early_)/(*I* ^DCE^_native_), where *I* ^DCE^_x_ was the mean T1w signal intensity in each respective phase. IE was categorized as fast when IE >1.5 or medium if IE <1.5. WO was categorized as persistent if >0.1, as plateau when between −0.1 and 0.1, and as wash-out if <−0.1 [27].
2. DWI: ADC maps generated using dedicated software (Syngo BreVis, Siemens, Erlangen, Germany) based on low and high *b* value DW images (*i.e.*, 50 and 850 s/mm^2^) and their average values were recorded.
3. T2w-MRI: average intensities of T2w images were recorded.
4. [^18^F]FMISO PET images: tumor-to-background (TBR) ratio was calculated by measuring the maximum and peak standard uptake value (SUV_max_ and SUV_peak_, respectively). SUV_peak_ was defined as mean SUV in a 1-cm^3^ sphere around SUV_max_. TBRs were defined as the respective SUV normalized to the mean SUV in 10 cm^3^ of gluteal muscle.

GTV volumes were recorded for the first three TPs and cervix volume was recorded at FU. To assess the temporal changes of the recorded parameters, the ratio of the change with respect to the previous imaging TP (X^TP-TP^) and to the BL scan (X^TP-BL^) was determined in percent as (value in TP_n_ − value in TP_n-1_)/(value in TP_n-1_) and (value in TP_n_ − value in BL)/(value in BL), respectively. To assess the spatio-temporal stability of hypoxia, the hypoxic regions were defined on the PET images from respective time-points (${\mathrm{ROI}}_{{\mathrm{TP}}_{\mathrm{n}}}$) and the overlaps between these regions were measured for neighboring time-points (${\mathrm{ROI}}_{\mathrm{TPn}}\cap {\kern0.2em \mathrm{ROI}}_{\mathrm{TPn}+1}$). The Sørensen–Dice coefficient was then calculated, patient-wise, for three time-point pairs (BL–TP1, TP1–TP2, and TP2–FU) using the following formula:

$$ \mathrm{DC}=\frac{2\left({\mathrm{ROI}}_{{\mathrm{TP}}_{\mathrm{n}}}\cap {\mathrm{ROI}}_{{\mathrm{TP}}_{\mathrm{n}+1}}\right)}{{\mathrm{ROI}}_{{\mathrm{TP}}_{\mathrm{n}}}+{\mathrm{ROI}}_{{\mathrm{TP}}_{\mathrm{n}+1}}} $$

### Voxel-by-Voxel Analysis

Additionally, the voxel-by-voxel analysis of all mentioned MRI and PET parameters on the delineated GTVs was performed using an in-house-developed MATLAB script. Great care was taken that no overlap of the GTV and bladder or rectum occurred. Only values between the 0.5th and 99.5th percentiles were taken into consideration. TBR ratio maps for PET, as well as IE and WO maps for DCE-MRI, were calculated according to the description above.

### Statistical Analysis

Statistical analyses were performed using the Statistical Package for the Social Sciences (IBM SPSS Statistics 22.0). Correlations between items were assessed using non-parametric Spearman’s rank correlation coefficient. *P* values <0.05 were considered significant (a two-tailed significance test was used). To assess the significance of a parameter’s change between the respective time-points, a pairwise Student’s *t* test with Bonferroni’s correction was used.

Correlations of voxel-by-voxel analysis between the imaging parameters were assessed with the non-parametric Spearman’s rank correlation coefficient and by plotting scatter plots for each patient, which combined each pair of investigated parameters. To compare quantitative mp-MRI information with PET, only [^18^F]FMISO PET voxels with a TBR higher than 1.4 were taken into account (TBR_1.4_).

## Results

### Histology and Tumor Size

All patients included had histologically confirmed squamous cell carcinoma of the cervix, stages IIB–IIIB. The mean intervals from treatment start, TP1, TP2, and FU imaging were 18 days (range, 13–19 days), 34 days (range, 27–35 days), and 132 days (range, 125–148 days), respectively. Median GTV volumes were 68.2 ± 31.1 cm^3^ at BL, decreased to 39.3 ± 18.8 cm^3^ at TP1, to 25.7 ± 15.1 cm^3^ at TP2, and the post-treatment cervix volume was 13.2 ± 6.0 cm^3^ at FU.

### Diffusion-Weighted MRI

The median tumor ADCs (in ×10^−3^ mm^2^/s) were 0.99 ± 0.10 (range, 0.79–1.10) at BL and increased at each imaging session to 1.20 ± 0.12 (range, 1.06–1.37) at TP1, to 1.33 ± 0.14 (range, 1.00–1.43) at TP2, and to 1.38 ± 0.21 (range, 1.30–1.51) at FU. The median ADC values in the reference structure muscle did not vary systematically between the different TP. The average ADC increase in the tumor reached statistical significance at all TP with respect to BL.

### Contrast-Enhanced MRI

As far as the curve type analysis of the DCE-MRI is concerned, at BL, five patients showed a medium IE and one fast IE. This reversed at TP1, where five patients showed a fast IE and one showed a medium IE. The patient with medium IE at BL remained medium, while one patient changed category. At TP2, all patients showed a fast IE, while after therapy, at FU, all patients but one had a medium IE (average dropped by 14.3 %). This patient also showed a medium IE at BL. The WO characteristics were as follows: at BL, four patients presented wash-out type signal intensity kinetics and two showed plateau curve types. At TP1, five patients showed wash-out and one showed plateau characteristics, but four patients changed categories, compared to BL. At TP2, again, five patients showed wash-out and one showed plateau characteristics, with two patients changing categories, compared to TP1. At FU, all patients presented with persistent enhancement.

### [^18^F]FMISO PET

For the [^18^F]FMISO scans, the median TBR_peak_ was 2.7 ± 0.8 (range, 2.0–4.6) at BL and decreased as follows: to 1.6 ± 0.2 (range, 1.5–2.1) at TP1, to 1.8 ± 0.3 (range, 1.2–2.3) at TP2, and to 1.7 ± 0.3 (range, 1.4–2.1) at FU. The decrease in TBR_peak_ was statistically significant only when assessed with respect to BL. The trend toward changes observed for SUV_max_ was of the same magnitude as those for SUV_peak_.

**Fig. 1:**
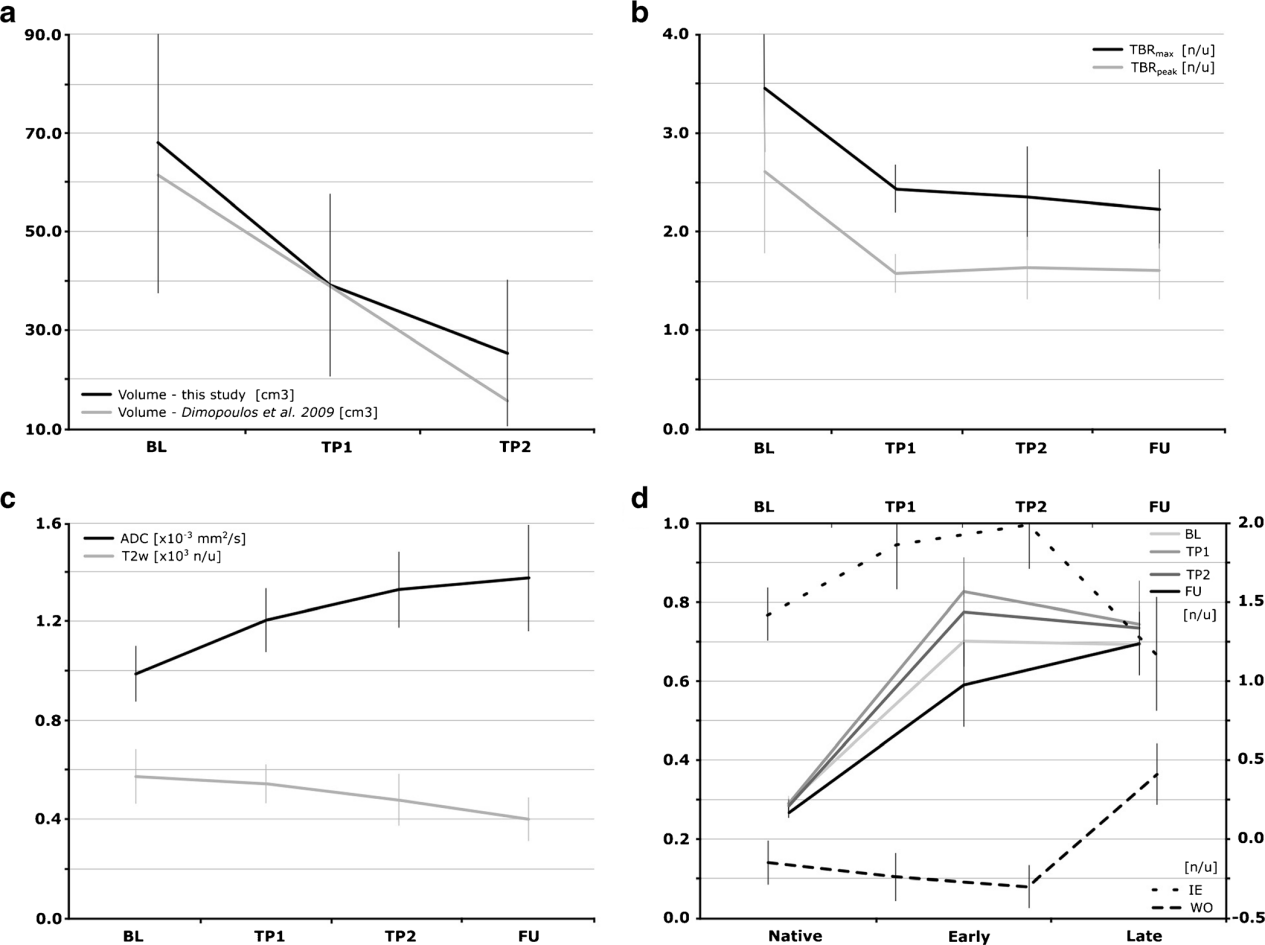
Median values of GTV volume and various imaging parameters including standard deviation at each time-point. **a** Tumor volume in comparison to ref. [30]. **b** TBRs. **c** T2w intensity and ADC. **d** IE and WO as well as enhancement curves of the T1w imaging with contrast (native, early, and late phases). BL, TP1, TP2, and FU—scan time-points: baseline, 2 and 5 weeks after EBRT start, follow-up (week 19); *TBR*_*max*_ (SUV_max_ in GTV)/(SUV_mean_ in gluteal muscle), *TBR*_*peak*_ (SUV_peak_ in GTV)/(SUV_mean_ in gluteal muscle); *GTV* gross tumor volume, *SUV*_*peak*_ standard uptake value measured in a 1-cm^3^ sphere around SUV_max_, *ADC* apparent diffusion coefficient, *T2w* T2 weighted MRI, *IE* initial enhancement defined as (*I* ^DCE^_early_ − *I* ^DCE^_native_)/(*I* ^DCE^_native_), *WO* wash-out rate defined as (*I* ^DCE^_delayed_ − *I* ^DCE^_early_)/(*I* ^DCE^_native_).

**Fig. 2:**
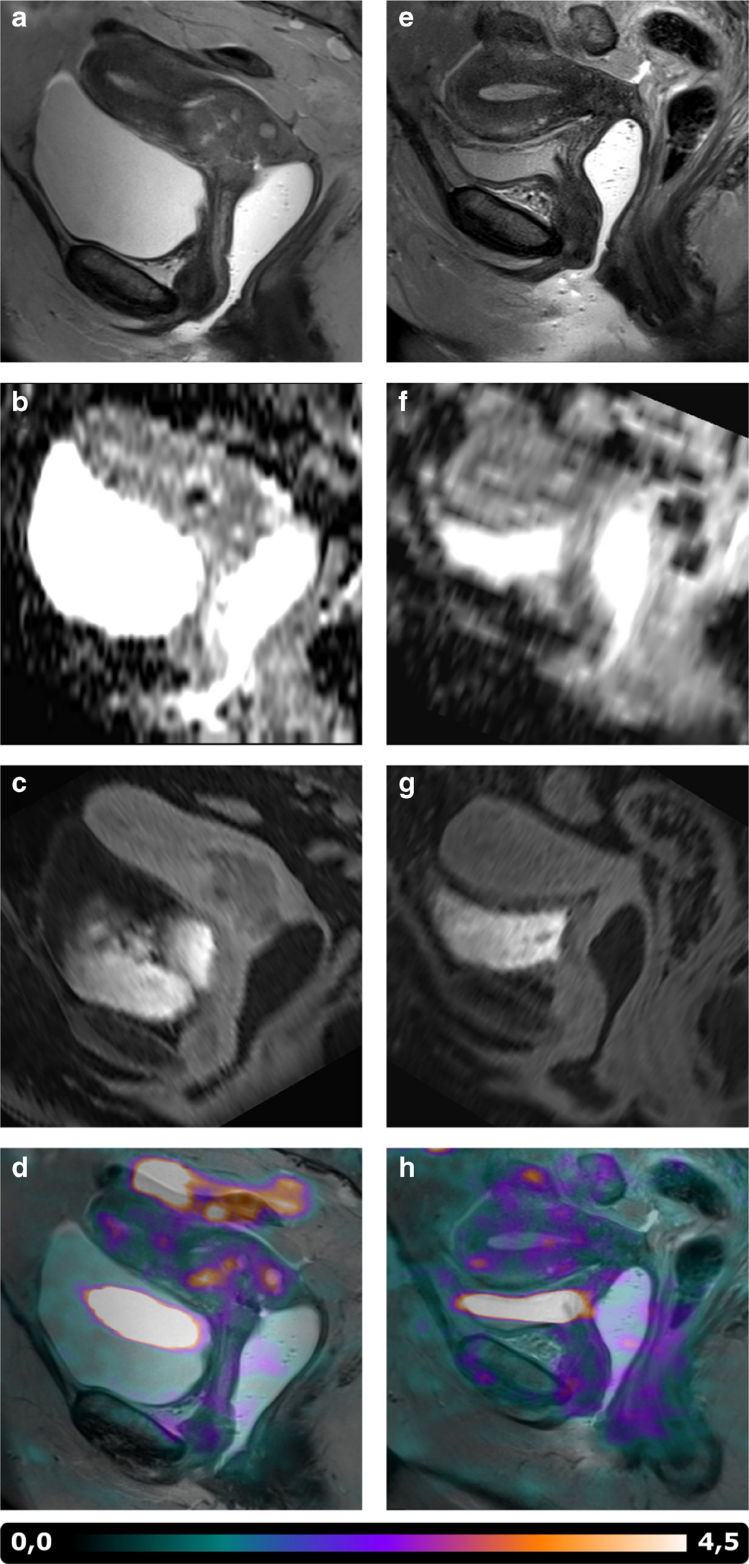
MR and PET images of a representative patient at **a**–**d** baseline and **e**–**h** follow-up. **a** and **e** show the decrease in tumor volume, and **b** and **f** show the respective increase of ADC values. **c** and **g** show the disappearance of wash-out regions on late DCE MR, and **d** and **h** visualize the decrease in [^18^F]MISO uptake.

Temporal changes for all recorded values are visualized in Fig. 1 and summarized in Table 3. As an example, Fig. 2 illustrates MR and PET images of a representative patient at BL and FU.

**Table 3:** Summary of various patient-related parameters at different time-points and temporal changes with respect to previous time-points and baseline investigations

	BL			TP1			TP2			FU		
Tumor volume (cm^3^)	68.2	±	31.1	39.3	±	18.8	25.7	±	15.1	13.2^a^	±	6.0
Volume^TP-TP^ (%)		N/A		−43	±	29	−42^p^	±	26		N/A	
Volume^BL^ (%)		N/A		−43	±	29	−72^p^	±	23		N/A	
TBR_max_	3.4	±	0.9	2.4	±	0.3	2.3	±	0.5	2.2	±	0.5
TBR_max_^TP-TP^ (%)		N/A		−34^p^	±	12	−3	±	14	−14	±	15
TBR_max_^BL^ (%)		N/A		−34^p^	±	12	−33^p^	±	12	−32^p^	±	9
TBR_peak_	2.6	±	0.8	1.6	±	0.2	1.7	±	0.3	1.6	±	0.3
TBR_peak_^TP-TP^ (%)		N/A		−29^p^	±	12	0	±	14	4	±	15
TBR_peak_^BL^ (%)		N/A		−29^p^	±	12	−34^p^	±	8	−321^p^	±	13
T2w	573.0	±	101.5	543.0	±	72.4	477.5	±	96.3	398.5	±	80.9
T2w^TP-TP^ (%)		N/A		−6	±	6	−8	±	9	−14	±	12
T2w^BL^ (%)		N/A		−6	±	6	−13	±	8	−27^p^	±	15
ADC (×10^−3^ mm^2^/s)	0.99	±	0.10	1.21	±	0.12	1.33	±	0.14	1.38	±	0.22
ADC^TP-TP^ (%)		N/A		22^p^	±	8	7	±	10	3	±	7
ADC^BL^ (%)		N/A		22^p^	±	8	35^p^	±	20	41^p^	±	24
IE^b^	1.4	±	0.2	1.9	±	0.3	2.0	±	0.4	1.2	±	0.4
IE^TP-TP^ (%)^c^		N/A			N/A^p^			N/A			N/A^p^	
IE^BL^ (%)^c^		N/A			N/A^p^			N/A			N/A^p^	
WO^b^	−0.2	±	0.1	−0.2	±	0.2	−0.3	±	0.1	0.4	±	0.2
WO^TP-TP^ (%)^c^		N/A			N/A			N/A			N/A^p^	
WO^BL^ (%)^c^		N/A			N/A			N/A			N/A^p^	

*TBR*_*max*_ (SUV_max_ in GTV)/(SUV_mean_ in gluteal muscle), *TBR*_*peak*_ (SUV_peak_ in GTV)/(SUV_mean_ in gluteal muscle), *SUV*_*peak*_ SUV measured in a 1-cm^3^ sphere around SUV_max_, *X*_*TP-TP*_ (value in TP_n_ − value in TP_n-1_)/(value in TP_n-1_), *X*_*BL*_ (value in TP_n_ − value in _BL_)/(value in _BL_)

^a^Volume of cervix at FU

^b^IE/WO calculated based on mean values of T1w _native/early/late_

^c^Not possible to calculate difference in ratios due to very low denominator value, only significance of change was assessed

^p^Stat. significant (*p* < 0.05)

The PET-based temporal stability of hypoxia assessment showed that the patient averaged DC values were 19.0, 14.0, and 9.4 %, respectively, for BL–TP1, TP1–TP2, and TP2–FU. The respective ranges were 0 to 53.9 %, 0 to 51.8 %, and 0 to 35.2 %.

### Voxel-by-Voxel Analysis

**Fig. 3:**
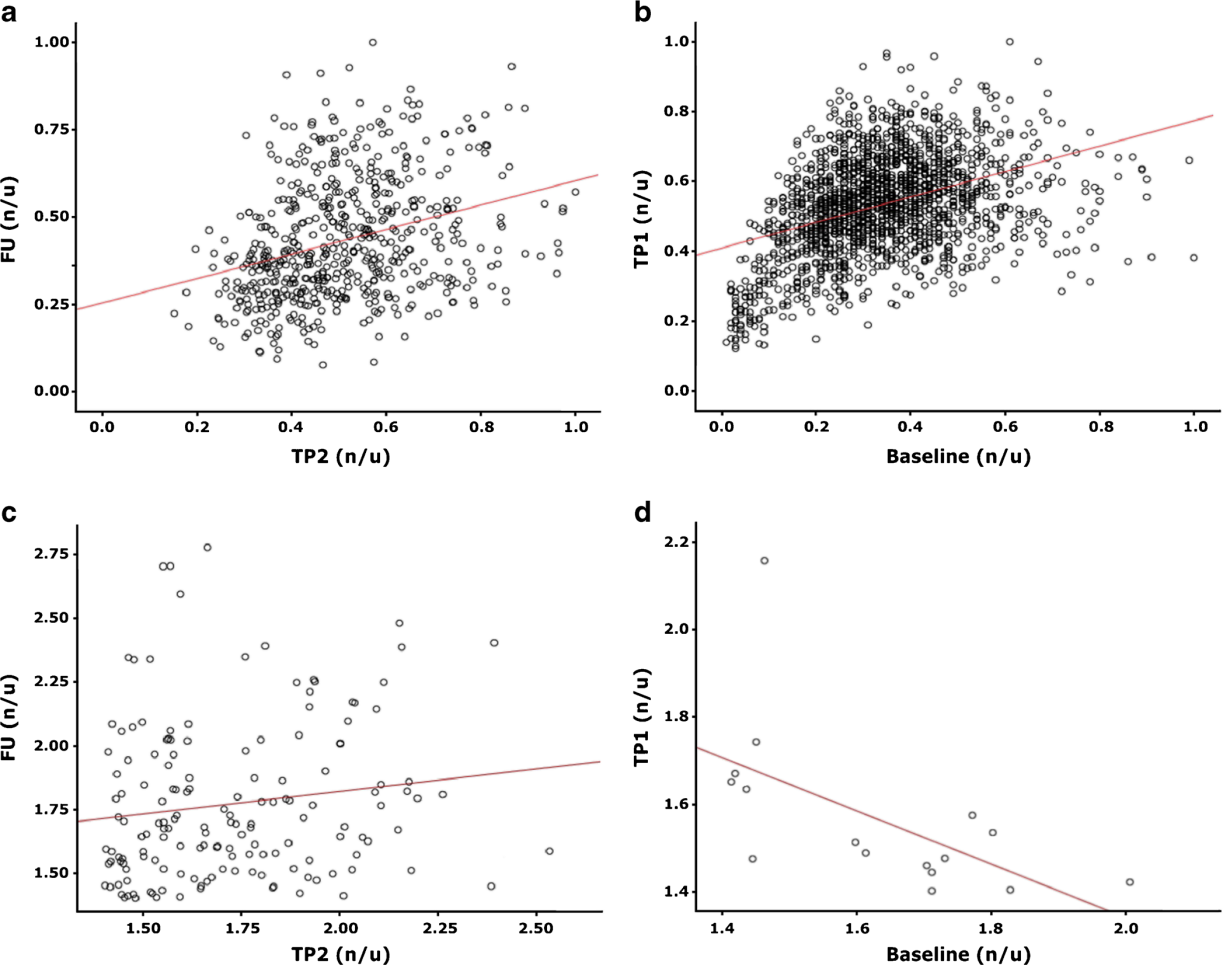
Scatter plots from different patients showing the correlation between SUV values determined in the GTV in two neighboring time-points. **a**–**b** Normalized to SUV_max_ at the respective time-point. **c**–**d** TBR limited to voxels with TBR >1.4. **a** Correlation of 0.34 (patient 6). **b** Correlation of 0.38 (patient 2). **c** Correlation of 0.16 (patient 6). **d** Correlation of −0.63 (patient 3). TP1, TP2, and FU—scan time-points: 2 and 5 weeks after EBRT start, follow-up (week 19); *SUV* standard uptake value, *GTV* gross tumor volume, *TBR* (SUV in GTV)/(SUV_mean_ in gluteal muscle).

The additionally performed voxel-by-voxel analysis of the [^18^F]FMISO scans at BL and TP1 showed no correlation. For TP2 and TP1 and FU and TP2, a weak correlation was found only for one patient at the respective TP, but for a different patients in each case. When only voxels with TBR >1.4 were taken into account just one, strong, but negative correlation was found between TP1 and BL, indicating the instability of hypoxic regions. Figure 3 shows examples of respective scatter plots.

The voxel-by-voxel analysis between the PET and MRI parameters revealed only one significant correlation of TBR_1.4_ and ADC in one patient at BL. No statistically significant correlations were found at TP1, TP2, and in the FU.

## Discussion

With the goal of achieving personalized treatment strategies, the well-known inter- and intra-tumor heterogeneity and the upcoming advanced imaging technologies have stimulated various researches on tumor characterization, treatment response assessment, and outcome prediction [1–10, 12–14, 16–18]. Consequently, dose painting strategies that aim to overcome treatment resistance by locally increasing the dose to the potentially more radio-resistant target sub-regions have been conceptually proposed and have been clinically introduced in selected studies for lung and head and neck cancer [11, 15, 19, 21, 22, 31]. Multimodality and multiparametric imaging is, from a theoretical point of view, the ideal method to investigate three important components of the tumor microenvironment: perfusion, diffusion, and hypoxia. However, the change in these parameters over the whole treatment course and beyond is not known. We aimed to investigate the spatio-temporal stability of tumor characteristics in cervix cancer patients using serial mp-MRI and hypoxia PET to evaluate subsequent image correlation. Although image registrations were performed with great care, it has to be pointed out that the accuracy and robustness of the deformable registration algorithms adds systematic uncertainties to the evaluation, especially with respect to tumor volume changes. However, this is a general research issue in response assessment, longitudinal studies in radiation oncology.

According to our knowledge, the current study is the first one to report [^18^F]FMISO results for cervix cancer patients. By analyzing the extent of the overlap in hypoxic regions between the recorded time-points, we could demonstrate that the topographic location of hypoxic subvolumes, as assessed by [^18^F]FMISO PET, are not stable during the treatment course. This is in line with the results of the voxel-by-voxel analysis. In our small sample size, we provide for the first time evidence that the spatio-temporal stability of hypoxia is typically non-existent in cervix cancer, and therefore dose painting strategies cannot be easily adopted. Although this observation is different from what others have reported about hypoxia in head and neck or in lung tumors (*cf.* [9, 19, 32, 33]), it confirms the possible reported variation between patients in terms of its stability [19]. Analysis based on larger patient groups is necessary to stratify the patient subgroups that might benefit from dose boosting in hypoxic regions. The TBR_max_ values were comparable to values typically reported for head and neck cancer, confirming the presence of hypoxia in this tumor histology [32, 33]. The ADC values and DCE parameters, as well as the changes over time observed in our study, are in agreement with values reported by others [8]. Six of seven evaluated patients had a complete local response to the treatment, and the magnitude of the changes over time was similar to volumetric response assessments by others [34] (*cf.* Table 1). The patient with local recurrence (P3) showed the smallest drop in TBR_max_ when comparing FMISO at BL and TP1 (11 % *vs.* median 35 % for the other patients) and BL *vs.* TP2 (14 % *vs.* 38 % for other patients). This might indicate persistent tumor hypoxia. However, this patient also developed distant metastasis.

The voxel-by-voxel analysis between MRI and PET, which was based on a TBR higher than 1.4, was performed similar to other studies [18, 27]. Missing correlations suggest that for detailed information about tumor perfusion, cell density, and hypoxia, complementary imaging modalities are needed and cannot be easily replaced.

The workload related to the presented analysis is significant and might be difficult to implement in other departments. Automation of related tasks through the use of scriptable software tools, and imaging performed on a hybrid scanner, can significantly reduce the workload and the resources necessary to conduct such longitudinal imaging studies.

The major limitation of our study is the small sample size. Although the [^18^F]FMISO PET-MRI performed in the absence of a hybrid scanner is feasible from a technical point of view for BL imaging, there are severe limitations for longitudinal response assessment studies. Patient compliance is a major factor as we lost 60 % of our patients due to medical reasons or due to demanding and time-consuming imaging procedures in addition to the intensive CBCT-guided IMRT and MRI-guided brachytherapy. This imaging study has been transferred to a recently installed hybrid PET/MR scanner, where we observed much higher patient tolerance. Moreover, simultaneous MRI and PET imaging overcomes logistic challenges between the imaging and radiotherapy departments, inaccuracies in patient positioning, image fusion, image post-processing, and data correlation, respectively. Deformable registration in our study represents a limitation that can in particular cases exceed the used voxel size, which can be avoided (intra time-point) by using a hybrid scanner.

## Conclusions

In conclusion, longitudinal, multiparametric MRI-PET imaging is feasible and provides complementary information about tumor characteristics. Serial multimodality imaging is workload intensive and demanding for patients. The hybrid PET/MR scanner seems to be an ideal tool for multiparametric response assessment studies in radiation oncology, although its place in research is a controversial issue [35]. Despite the need for more personalized treatment approaches to improve the therapeutic ratio, especially in radio-resistant tumors, developing dose painting treatment strategies should be carefully adapted according to tumor biology and the expected performance during the treatment course.
